# Clear cell tumor of the lung could be aggressive: a case report and review of the literature

**DOI:** 10.1186/s13019-020-01224-w

**Published:** 2020-07-20

**Authors:** Leilei Shen, Jixing Lin, Zhipeng Ren, Bailin Wang, You Liu, Jing Yuan, Lianbin Zhan

**Affiliations:** 1 Department of Thoracic Surgery, Hainan Branch of PLA General Hospital, Sanya, 572013 China; 2 Department of Pathology, Hainan Branch of PLA General Hospital, Sanya, China

**Keywords:** Clear cell tumor of the lung, Sugar tumor, Immunohistochemical analysis, Multiple, Aggressive

## Abstract

**Background:**

Clear cell tumors of the lung (CCTLs) are rare and mostly benign pulmonary neoplasms arising from perivascular epithelioid cells. Only approximately 100 cases have been reported, and half of them were in China. Limited details about CCTLs often cause diagnostic or therapeutic problems.

**Case presentation:**

We describe a case of a 28-year-old woman with multiple gradually replicating and enlarging nodules in the left lower lobe. The patient underwent fine-needle aspiration biopsy and was diagnosed with CCTL. A left lower lobectomy and mediastinal lymph node dissection were performed. The gradual changes in size (1.4 cm to 2.8 cm) and quantity (10 to 49) of the CCTLs in this case were the biggest differences from previously reported cases.

**Conclusions:**

CCTLs are very uncommon and mostly benign PEComatous tumors with no specific morphologic features. We present a case of CCTL with multiplicity and rapid growth, which may indicate its aggressive nature. The accumulation of similar cases will help clarify the exact nature and improve our understanding of the disease.

## Background

Clear cell tumors of the lung (CCTLs) are extremely rare and benign lesions first described in 1963 by Liebow and Castlemen [1] in four patients. This type of tumor consists of thin cell walls and high levels of glycogen and shows positivity for periodic acid-Schiff (PAS) staining. Therefore, CCTLs have also been termed “sugar tumors” [2]. CCTLs belong to a family of tumors arising from putative perivascular epithelioid cells, called “PEComatous tumors, benign” of the lung, according to the 2015 World Health Organization (WHO) classification of lung tumors [3]. The tumor cells show immunoreactivity for S100 and HMB45 and no cytokeratin reactivity, which differentiates them from renal cell carcinoma and can be used to make a definitive diagnosis [4]. To date, fewer than 100 cases have been reported, and misdiagnosis occurs occasionally. Here, we report an interesting patient with multiple CCTLs to improve our understanding of the diversity of the disease.

## Case presentation

**Fig. 1:**
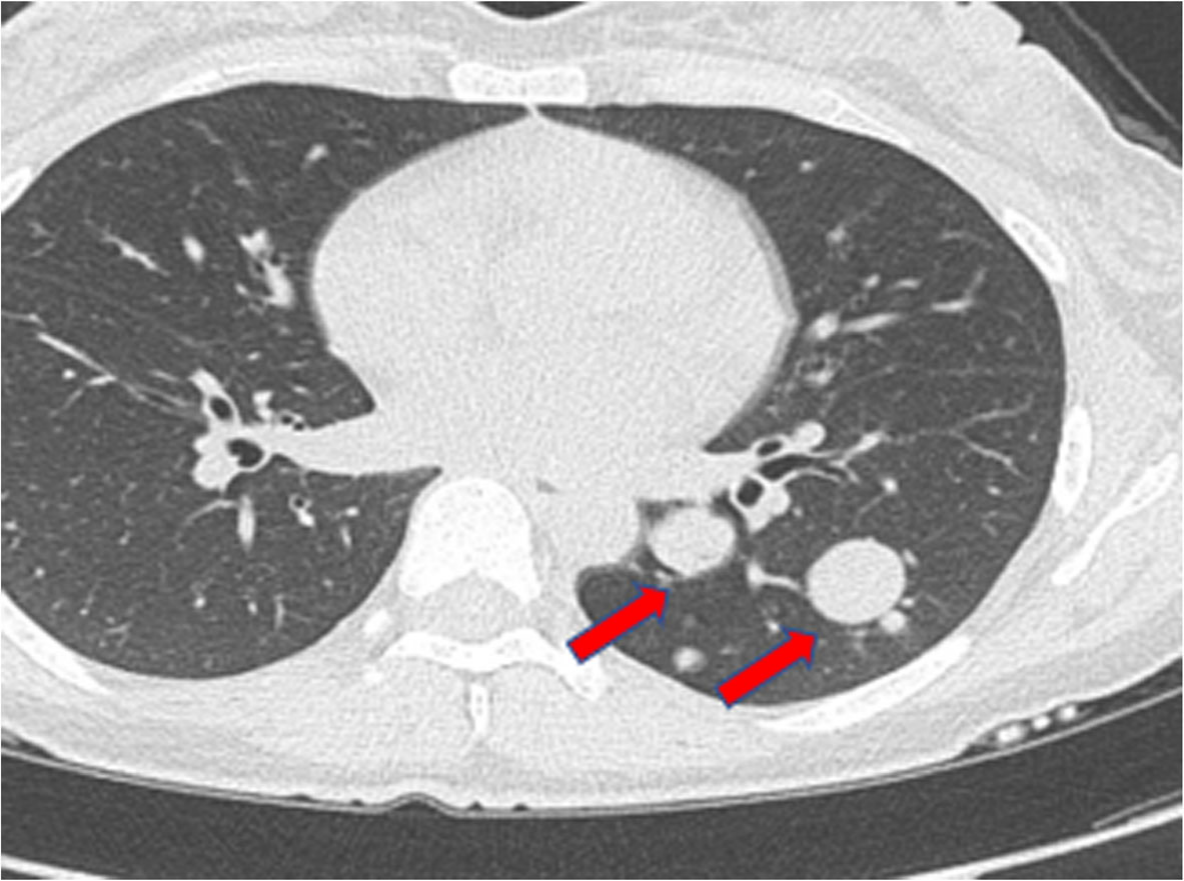
Chest CT showed multiple (approximately 49) nodules and a maximal 2.8 cm nodule in diameter in the left lower lobe

**Fig. 2:**
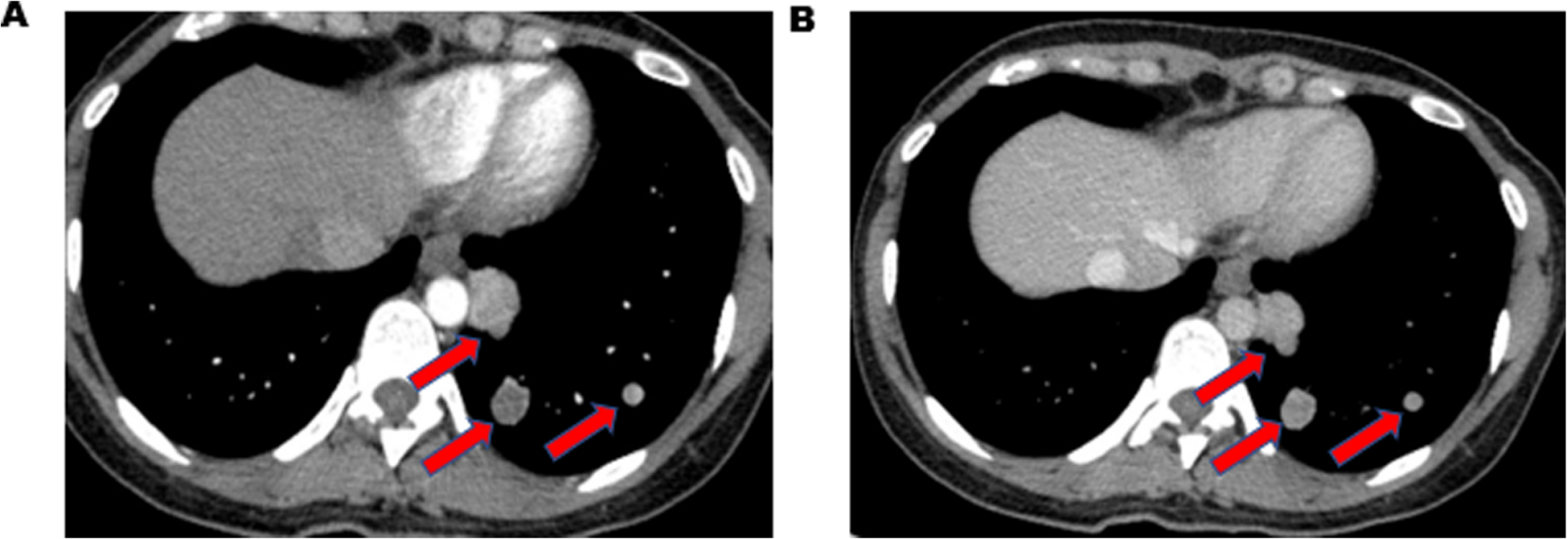
**a** chest CT revealed intense heterogeneous enhancement in the aterial phase. **b** chest CT showed nodules’ homogeneous nature in the delay phase

**Fig. 3:**
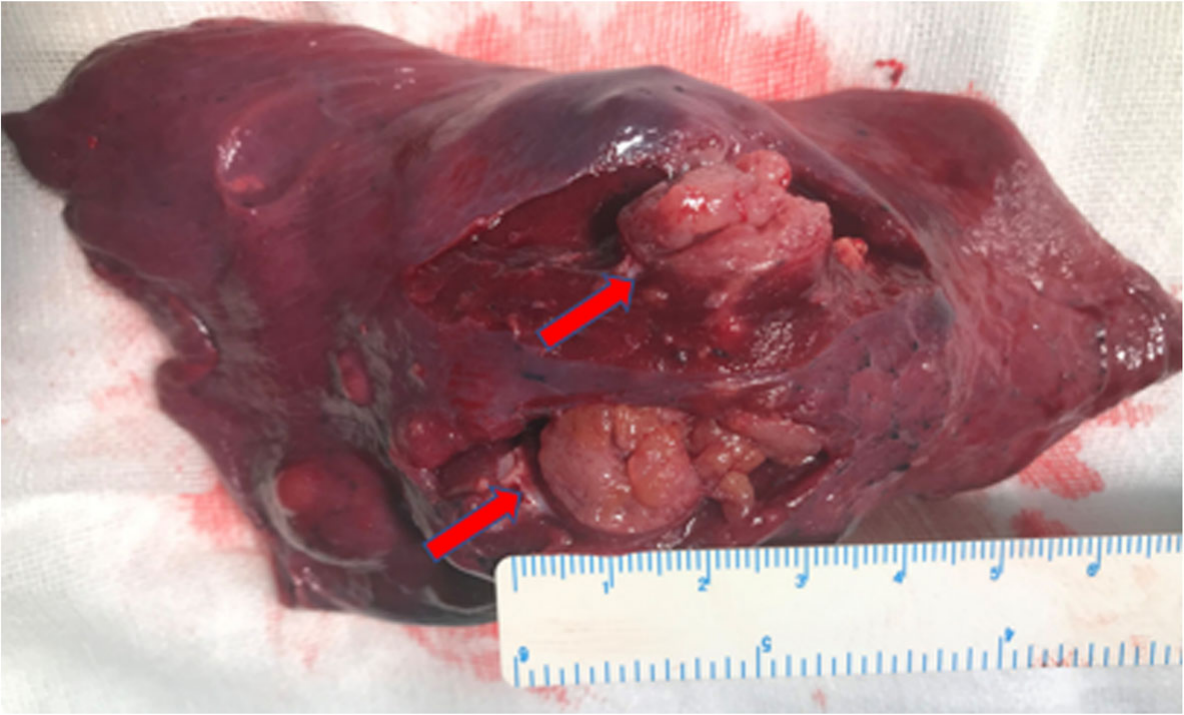
The tumor was solid, grey-white, with a soft texture and clear boundaries grossly

A 28-year-old woman consulted the hospital for left chest paroxysmal pricking in 2014. Chest plain computed tomography (CT) scans revealed approximately 10 demarcated, round, homogeneous, unequally sized nodules without evidence of calcification, necrosis, or cavitation in the left lower lobe. Regular follow-up was suggested, and the same CT findings were obtained in 2016. In March 2019, she was admitted to our hospital for further evaluation because a plain chest CT scan showed larger, more numerous nodules. There were approximately 49 nodules, the largest of which was 2.8 cm in diameter (Fig. 1). Interestingly, all nodules were in the left lower lobe. Contrast-enhanced arterial phase CT showed intense heterogeneous enhancement (Fig. 2a), and the delay phase showed homogenous nodules (Fig. 2b). CT-guided biopsy was performed to determine the nodule’s nature. Pathology indicated CCTL. No distal metastasis was found; therefore, left lower lobectomy and mediastinal lymph node dissection (stations 5, 7, 8, 9, 10, and 11) were performed. Postoperative pathological analysis confirmed the diagnosis of CCTL. Grossly, the tumor was solid, gray-white, with a soft texture and clear boundaries (Fig. 3). The largest CCTL nodule measured 2.8 cm.

**Fig. 4:**
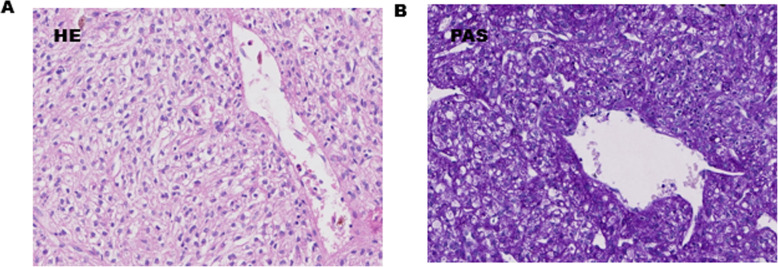
**a** HE staining (× 40) showed the tumor consisted of rounded clear cells with distinct cell borders and granular eosinophilic cytoplasm. **b** PAS staining (× 40) was positive

**Fig. 5:**
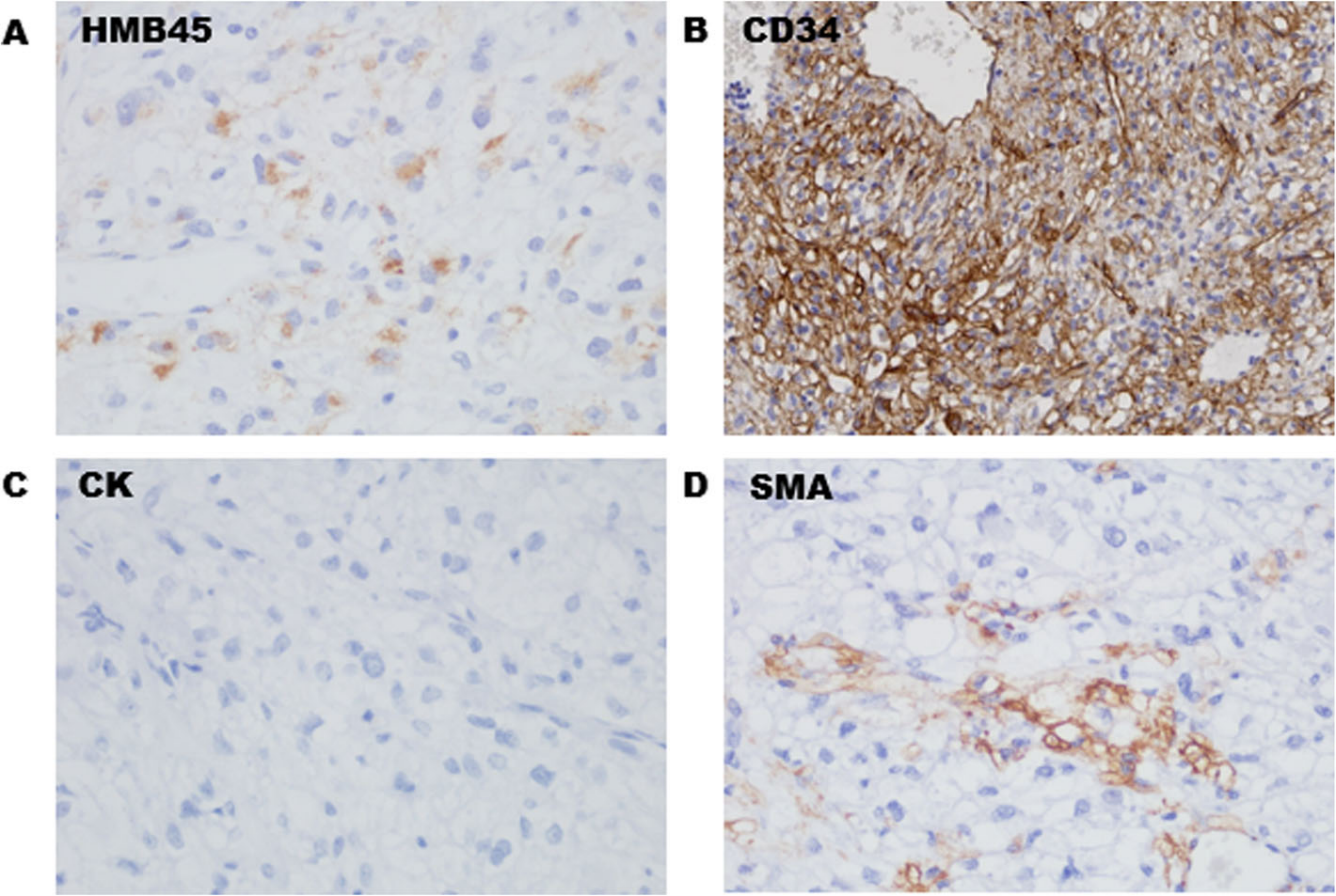
The immunohistochemical profile (× 40) showed positivity for HMB45(**a**) and CD34(**b**), while revealed negative reactivity for cytokeratin(**c**) and SMA(**d**)

HE staining showed that the tumor consisted of round, clear cells with distinct cell borders and a granular eosinophilic cytoplasm (Fig. 4a). Histology revealed cytoplasmic PAS-positive clear cells without atypia, mitosis, or necrosis (Fig. 4b). The immunohistochemical profile of the clear cells was positive for HMB45 (Fig. 5a), CD34 (Fig. 5b), and Vimentin. Tumor reactivity was negative for cytokeratin (Fig. 5c), SMA (Fig. 5d), S-100, CD10, PAX-8, desmin, and Myo-D1. No lymphatic metastasis was observed. The postoperative course was uneventful. No evidence of metastasis or recurrence was observed during the 6-month follow-up period after the surgery.

## Discussion

The present clinicopathologic study of CCTL, otherwise defined as “sugar tumor”, is based on a small number of sporadic reports. Limited details about CCTL may cause diagnostic or therapeutic problems. Therefore, every single case is essential for pathologists or surgeons to better understand the nature of CCTL.

CCTLs belongs to a family of “PEComatous tumors, benign” of the lung [3], and surgical excision with no need for adjuvant chemo- or radiotherapy is the main treatment. We can conclude that CCTL occurs at any age but presents with a high incidence in elderly individuals according to a review of the reported cases [5–9]. The disease can occur in either sex, but some researchers consider it to have a slight female predominance [9]. Often incidentally discovered by chest CT scans, CCTL commonly presents as a one or multiple rounded, smooth-walled peripheral parenchymal lesions with no evidence of cavitation or calcification. Most patients are asymptomatic, and Wang et al. [4] considered that tumor size may be related to the patient’s manifestations. CCTLs range in size from 1 mm [5] to 12 cm [6] and tend to produce symptoms such as chest pain, cough, breathlessness, hemoptysis, or fever [8–13] more frequently when they are ≥2.2 cm. In our report, the patient experienced symptoms of chest pricking, and the largest nodule was 2.8 cm in diameter.

Radiographically, malignancy cannot be excluded based on CT findings alone, and CCTLs are often considered primary or metastatic lung cancer due to the rich vascular stroma revealed by intense postcontrast enhancement on CT findings [4]. However, knowledge concerning the features of CCTLs can be obtained from the intense heterogeneous enhancement in the arterial phase and homogeneous enhancement in the delay phase. No specific lobar distributions have been found, but the nodules generally affect the lower lobes of both lungs [7, 8]. In our case, there were approximately 49 nodules, all in the left lower lobe. CCTL is often diagnosed by lobectomy, segmentectomy, and wedge resection [14]. Occasionally, transbronchial lung biopsy [15] and fine-needle aspiration biopsy [9] are performed preoperatively to confirm the nature of the lesion. Some patients choose the “watch and wait” strategy.

Histologically, the lesions consist of round or oval clear cells with distinct cell borders and a granular eosinophilic cytoplasm. The characteristic feature of the lesions is a scanty intervening stroma with thin-walled sinusoidal vessels [9]. Strong positivity for PAS staining defined the lesion as a “sugar tumor” [2]. Mitosis is usually absent, and necrosis is extremely rare. Most immunohistochemical studies presented in previous reports [4, 16, 17] have shown unique immunoreactivity for HMB45 and CD34 but no reactivity for cytokeratin and desmin. CCTLs may also stain for S-100. It should be differentiated from other malignant clear cell tumors, including a clear cell variant of bronchogenic carcinoma and metastatic renal cell carcinoma. The immunoreactivity with cytokeratin helps differentiate these diseases from CCTL [14].

Biologically, CCTLs have traditionally been considered benign, but we report this interesting case to question the benign nature of CCTLs because of the multiplicity and rapid increase in size of the lesions. The clinicopathological features were consistent with those of the described cases. We analyzed 5 nodules and did not find vascular invasion or permeation of the tumor cells, and there were no histological differences among these 5 nodules. It is unclear whether the lesions were the result of intrapulmonary metastases or consisted of multiple primary lesions. Approximately 100 CCTL cases have been reported to date, and most of the nodules did not show malignant behavior. What would be the destiny of these patients if they did not undergo surgery? We speculate that patients with solitary CCTLs may experience long-term survival. However, surgical resection with VATS is a safe and curative approach, and we should not accept the low risk of local growth and distant metastasis. Whether or not surgery should be performed remains unclear because we seldom diagnose such tumors preoperatively and have not performed the RCTs necessary to answer this question. In 1988, we reported on a CCTL patient who died from hepatic metastases 10 years after resection [18], while two other reports described this tumor as having rapid growth [2, 19]. Kavunka et al. [6] also reported malignant behavior according to the observed vascularity and local invasion. Given these data, a left lower lobectomy and mediastinal lymph node dissection were performed, and long-term follow-up will be necessary.

## Conclusion

CCTLs are very uncommon and mostly benign PEComatous tumors with no specific morphologic features. We present a case of CCTL with multiplicity and rapid growth, which may indicate its aggressive nature. The accumulation of similar cases will help clarify the exact nature and improve our understanding of this disease.

## Data Availability

Please contact the corresponding author to request this information.

## Abbreviations

CCTL: Clear cell tumor of the lung
WHO: World Health Organization
PAS: Periodic acid-Schiff
CT: Computed tomography

## References

[1] Liebow AA, Castleman B (1963). Benign “clear cell tumors” of the lung. Am J Pathol.

[2] Liebow AA, Castleman B (1971). Benign clear cell “sugar” tumors of the lung. Yale J Biol Med.

[3] Travis WD, Brambilla E, Nicholson AG (2015). The 2015 World Health Organization classification of lung tumors: impact of genetic, clinical, and radiologic advances since the 2004 classification. J Thorac Oncol.

[4] Wang GX, Zhang D, Dia XW (2013). Clear cell tumor of the lung: a case report and literature review. World J Surg Oncol.

[5] Cavazza A, Sgarbi G, Ferrari G (2001). Clear-cell tumor of the lung: description of a case 1 mm in diameter (“micro-sugar tumor”). Pathologica.

[6] Kavunka AM, Pandiyan MS, Phili MA (2007). Large clear cell tumor of the lung mimicking malignant behavior. Ann Thorac Surg.

[7] Han B, Jiang G, Wang H (2010). Benign clear cell tumor of the lung. Ann Thorac Surg.

[8] Santana AN, Nunes FS, Ho N (2004). A rare cause of hemoptysis: benign sugar (clear) cell tumor of the lung. Eur J Cardiothorac Surg.

[9] Policarpio-Nicolas ML, Covell J, Bregman S (2008). Fine needle aspiration cytology of clear cell “sugar” tumor (PEComa) of the lung: report of a case. Diagn Cytopathol.

[10] Gora-Gebka M, Liberek A, Bako W (2006). The “sugar” clear cell tumor of the lung-clinical presentation and diagnostic difficulties of an unusual lung tumor in youth. J Pediatr Surg.

[11] Zarbis N, Barth TF, Blumstein NM (2007). Pecoma of the lung: a benign tumor with extensive 18F-2-deoxy-D-glucose uptake. Interact Cardiovasc Thorac Surg.

[12] Sen S, Senturk E, Kuman NK (2009). PEComa (clear cell “sugar” tumor) of the lung: a benign tumor that presented with thrombocytosis. Ann Thorac Surg.

[13] Yan B, Yau EX, Petersson F (2011). Clear cell “sugar” tumor of the lung with malignant histological features and melanin pigmentation - the first reported case. Histopathology.

[14] Hirata T, Otani T, Minamiguchi S (2006). Clear cell tumor of the lung. Int J Clin Oncol.

[15] Takanami I, Kodaira S, Imamura T (1998). The use of transbronchial lung biopsy to establish a diagnosis of benign clear cell tumor of the lung: report of a case. Surg Today.

[16] Adachi Y, Kitamura Y, Nakamura H (2006). Benign clear (sugar) cell tumor of the lung with CD1a expression. Pathol Int.

[17] Tsilimigras DI, Bakopoulos A, Ntanasis-Stathopoulos I (2018). Clear cell “sugar tumor” of the lung: diagnostic features of a rare pulmonary tumor. Respir Med Case Rep.

[18] Sale GE, Kulander BG (1988). “Benign” clear-cell tumor (sugar tumor) of the lung with hepatic metastases ten years after resection of pulmonary primary tumor. Arch Pathol Lab Med.

[19] Kalkanis A, Trianti M, Psathakis K (2011). A clear cell tumor of the lung presenting as a rapidly growing coin lesion: is it really a benign tumor?. Ann Thorac Surg.

